# Termination of pregnancy and sterilisation in women with childhood-onset type 1 diabetes

**DOI:** 10.1007/s00125-017-4428-7

**Published:** 2017-09-11

**Authors:** Lena Sjöberg, Risto Kaaja, Mika Gissler, Jaakko Tuomilehto, Aila Tiitinen, Janne Pitkäniemi

**Affiliations:** 1 Department of Public Health, University of Helsinki, Helsinki, Finland; 2 Department of General Practice and Primary Health Care, University of Helsinki, PO Box 20, 00014 Helsinki, Finland; 3 Chronic Disease Prevention Unit, National Institute for Health and Welfare, Helsinki, Finland; 4 Department of Internal Medicine, Institute of Clinical Medicine, University of Turku, Turku, Finland; 5 Turku University Hospital, Turku, Finland; 6 Information Services Department, National Institute for Health and Welfare, Helsinki, Finland; 7 Department of Neurobiology, Care Sciences and Society, Division of Family Medicine, Karolinska Institutet, SE-171 77 Stockholm, Sweden; 8 Dasman Diabetes Institute, Dasman, Kuwait; 9 Diabetes Research Group, King Abdulaziz University, Jeddah, Saudi Arabia; 10 Department of Obstetrics and Gynecology, University of Helsinki, Helsinki, Finland; 11 Department of Obstetrics and Gynecology, Helsinki University Hospital, Helsinki, Finland; 12 Finnish Cancer Registry, Helsinki, Finland

**Keywords:** Diabetes mellitus type 1, Epidemiology, Human, Pregnancy, Sterilisation, reproductive, Termination of pregnancy

## Abstract

**Aims/hypothesis:**

The aim of this study was to explore the association between type 1 diabetes and reproductive health indicators in women, focusing on termination of pregnancy and sterilisation.

**Methods:**

We conducted a registry-based cohort study involving 2281 women with childhood-onset type 1 diabetes, matched for age and birthplace with women without diabetes: two control participants for each woman with diabetes. We compared the frequencies of termination of pregnancy and sterilisation over a 25 year period between women with type 1 diabetes and women without, and estimated standardised incidence ratios (SIRs). Smoothed age and period effects in the incidence of termination of pregnancy or sterilisation were tested statistically.

**Results:**

There were more terminations of pregnancy (SIR 1.67; 95% CI 1.51, 1.86) and sterilisations (SIR 1.69; 95% CI 1.56, 1.83) in women with diabetes than in control women. During recent years, sterilisations in women with diabetes have decreased and the difference compared with control women has vanished. The indications for both procedures showed a statistically highly significant difference: maternal medical indications were almost absent (< 1%) in procedures among control women, but comprised 23.6% of terminations of pregnancy and 22.9% of sterilisations in women with diabetes.

**Conclusions/interpretation:**

The indications for termination of pregnancy and sterilisation are different in women with diabetes compared with other women. Pregnancies in women with type 1 diabetes are still terminated more often than in women without diabetes, but the difference in sterilisation rates has disappeared during recent years.

**Electronic supplementary material:**

The online version of this article (10.1007/s00125-017-4428-7) contains peer-reviewed but unedited supplementary material, which is available to authorised users.

## Introduction

The pregnancies of women with type 1 diabetes have been a matter of concern for many decades. The main concerns have been adverse effects on health of the offspring and the burden of pregnancy on maternal health, both short and long term. The risk of birth defects in children is higher in pregnancies complicated by type 1 diabetes [1]; furthermore, children of parents with type 1 diabetes have an approximately tenfold risk of developing diabetes compared with children whose parents do not have the disease [2, 3]. The risk of pregnancy complications in women with diabetes is higher than in non-diabetic pregnancies [1]. The effect of pregnancy on the development and progression of maternal diabetic complications is not unambiguous. Several studies have shown that hypertensive pregnancy may accelerate worsening of both retinopathy and nephropathy [4], but uncomplicated pregnancy might not necessarily worsen the prognosis from a long-term perspective [4–9].

Many collaborative plans have been made to establish pre-pregnancy targets for women with type 1 diabetes to minimise health risks for the woman and fetus [10]. Termination of pregnancy might be recommended in early pregnancy if the risk of serious health issues is considered high for the fetus, and sterilisation might be recommended if the risks of any potential future pregnancy are considered too high.

The frequencies of termination and sterilisation in women with type 1 diabetes compared with women without diabetes have only been evaluated in a few studies. The studies on termination in women with type 1 diabetes all report the proportion of terminations among all pregnancies, which varies from 7% to 18% [11–15]. Of these, the proportion performed for medical reasons varies between 25% and 37%. Most of the studies in this area compare women with diabetes with women in the general population [13–15]. In these, the frequency of termination is higher in women with type 1 diabetes than in the general population and, if the termination indications are included, the proportion of pregnancy terminations for medical reasons (maternal or fetal) is higher in women with type 1 diabetes than in the general population.

There are only a few studies on the use of contraception by women with type 1 diabetes. According to a Danish questionnaire study from 1992, sterilisation was much more common in women with type 1 diabetes than in those without diabetes (23% vs 9%) [16]. A British registry study from 1999 also showed a statistically significant difference in the proportion of women undergoing sterilisation: 5.8% of women with type 1 diabetes compared with 3.7% of women without type 1 diabetes [17].

The aim of this study was to assess the association between type 1 diabetes and two aspects of reproductive health, termination of pregnancy and sterilisation, in women with childhood-onset type 1 diabetes through a registry-based study. We compared the frequencies of termination of pregnancy and sterilisation, their indications, time trends and birth-cohort effects in a cohort of Finnish women with type 1 diabetes and a group of control women without diabetes over a 25 year period.

## Methods

We have examined registry data for a large representative nationwide cohort of Finnish people with childhood-onset type 1 diabetes. This cohort was initially identified for a study on mortality of people with type 1 diabetes—the Diabetes Epidemiology Research International (DERI) group’s mortality study [18]. Details on the selection of the cohort and the control participants are available in the electronic supplementary material (ESM) methods.

The Medical Birth Register was established at the start of 1987, which was defined as the start of follow-up in the present work. In this study, we used only data on live births from the Medical Birth Register. In our study cohort, 62 women died before the start of follow-up, 34 women with type 1 diabetes and 28 women without. After the exclusions, the database we used comprised information from 2281 women with type 1 diabetes and 4509 control women.

Physicians in Finland are obliged to report, using specific data collection forms, all terminations of pregnancy and sterilisations within 1 month of having performed a procedure. The Register of Induced Abortions was established in 1983 and the Register of Sterilisations in 1987.

In this registry-based study, we used information from several nationwide sources available in Finland: the Social Insurance Institution’s Drug Register, the Hospital Discharge Register (HDR) and the Medical Birth Register of the National Institute for Health and Welfare (diagnoses and procedures, as well as perinatal data), the Register on Congenital Malformations, the Register on Induced Abortions and Sterilisations, the Population Register Centre and Statistics Finland.

Names, birth dates, personal identity codes and other data by which people could be identified were removed after data linkage. During the analyses, the investigators used an individual six-digit study code to identify the study participants. The decoding key was kept separate from the study material. In every step of the study, effort was made not to reveal the identities of the study participants, as the data contained information that was potentially very sensitive. All researchers involved complied with the data protection principles and ethics rules of the University of Helsinki and the National Institute for Health and Welfare. The ethical committees of the Department of Public Health at the University of Helsinki and of the National Institute of Health and Welfare approved the study.

There were 286 deaths among the women with type 1 diabetes and 119 deaths among the control women; 1724 live births (1695 birth events) were recorded in women with type 1 diabetes and 5692 live births (5612 birth events) in control women.

The primary outcomes of the study were termination of pregnancy and sterilisation. We report the risks (hazard ratios) of pregnancy termination and sterilisation by age and calendar date. In instances of multiple sterilisation events in one woman, we considered only the last procedure. The observation period for the incidence of termination of pregnancy and sterilisation started at 1 January 1987 and ended at the time of sterilisation, the age of 50, death or censoring (31 December 2011), whichever occurred first.

To account for the potential difference in the probability of becoming pregnant between women with and without type 1 diabetes, we estimated the risk of termination of pregnancy, where the period at risk of termination of pregnancy started at the estimated time of conception and ended at either the time of birth of a child or the time of termination of pregnancy. This avoided the potential bias introduced by including women without any offspring and non-pregnant person-years of women with offspring when estimating the risk of termination of pregnancy. Data on spontaneous abortions are not available; hence, this is the proportion of terminations of pregnancy among pregnancies ending in either childbirth or termination.

Differences in rates according to the age and calendar time were tested by using likelihood ratio tests (LRTs) comparing separate spline functions by group (type 1 diabetes, control participants) with single-spline functions, using the mgcv package in R [19]. In order to adjust for both age and calendar time differences in rates of termination of pregnancy and sterilisation, we estimated standardised incidence ratios (SIRs) using reference rates from non-diabetic women and obtained 95% CIs by using standard Poisson regression.

We divided the terminated pregnancies into three groups according to the time intervals used in the Finnish abortion legislation: under 12 weeks of gestation (when termination of pregnancy is easily accessible); 12–19 weeks of gestation (when termination of pregnancy is possible but has to be applied for from the national board); and 20 or more weeks of gestation (when termination of pregnancy is possible only if fetal risks have been diagnosed or the life of the mother is threatened; and it has to be applied for from the national board). The frequencies of indications of termination of pregnancy and sterilisation were compared between type 1 diabetes and control women using standard *χ* ^2^ tests for independent groups. The indications for termination of pregnancy were grouped into five categories: maternal risk; social indication; age/parity (age either under 17 or over 40 years/the woman had previously four or more children); suspected or diagnosed birth defect; and no indication marked on the application form (a few women only). In Finland, termination of pregnancy has been approved by law since 1950, termination because of social reasons was legalised in 1971 and terminations of pregnancy outside the health sector are considered to have stopped at the start of the 1970s, at the latest.

## Results

### Terminations of pregnancy

The number of terminations of pregnancy, women with terminated pregnancies, rates and SIRs for type 1 diabetes and control women are shown in Table 1. There were 344 terminations of pregnancy in women with type 1 diabetes (*n* = 1123) and 722 terminations of pregnancy performed in control women (*n* = 2905) during a maximum of 25 years of follow-up. On average, 25% of women with type 1 diabetes and 20% of control women who were pregnant between 1987 and 2011 had experienced at least one termination of pregnancy. The rate of termination of pregnancy was 277 per 1000 person-years of being pregnant in women with type 1 diabetes and 167 per 1000 person-years of being pregnant in control women. The risk of termination of pregnancy (SIR) was increased by 67% (95% CI 51%, 86%) among women with type 1 diabetes compared with control women. Among the women with type 1 diabetes, 17.1% of pregnancies were terminated and among control women, 11.5% of pregnancies were terminated.

**Table 1:** Termination of pregnancy and sterilisation in women with and without type 1 diabetes

Variable	Termination of pregnancy		Sterilisation	
	T1D	Non-T1D	T1D	Non-T1D
Number of:				
Women at risk	1123	2905	2281	4509
Procedures	344	722	597	873
Births	1673	5535		
Person-years	1243	4317	41,308	92,328
Rate^a,b^	277^a^	167^a^	14.5^b^	9.5^b^
SIR^c^ (95% CI)	1.67 (1.51, 1.86)	Ref.	1.69 (1.56, 1.83)	Ref.

The control group are women without type 1 diabetes

^a^Terminations of pregnancy per 1000 pregnancy-years

^b^Sterilisation procedures per 1000 person-years

^c^Estimated by comparing age-period specific rates in women with type 1 diabetes with the comparable rates in the control group (reference group)

Ref., reference; T1D, type 1 diabetes

**Fig. 1:**
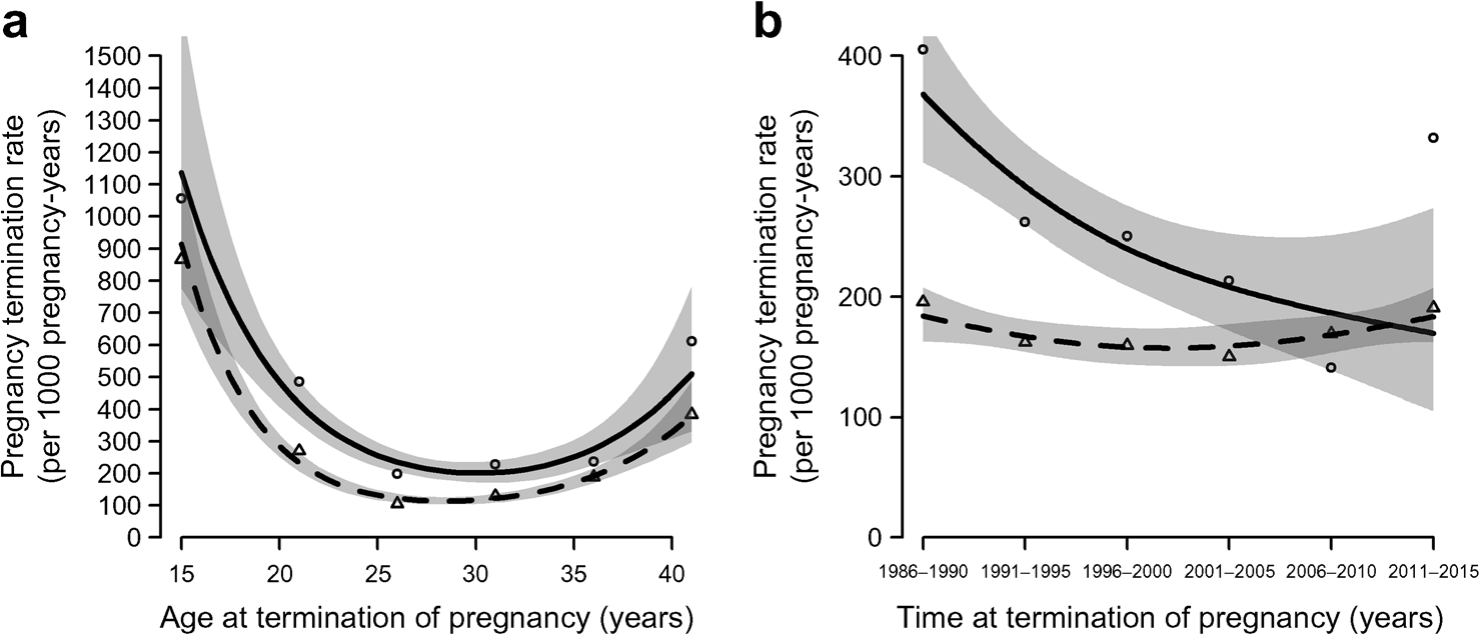
Pregnancy termination rates per 1000 pregnancy-years: (**a**) by age (*p* = 0.38); and (**b**) by calendar time (*p* = 0.11). The *p* values are for the difference between women with and without diabetes; 95% CIs are shown as shading. Solid line with circles, with type 1 diabetes; dashed line with triangles, without type 1 diabetes

The risk of termination of pregnancy did not vary by age between women with type 1 diabetes and control women (*p* = 0.38, Fig. 1). Slightly more terminations were performed among women with type 1 diabetes than in those without in women who became pregnant under the age of 30.

Comparing hazard ratios in the two groups of women for terminations in relation to calendar time, no statistically significant difference was seen (*p* = 0.11, Fig. 1); more terminations of pregnancy were observed among women with type 1 diabetes than in control women before 1995.

In women with type 1 diabetes, the terminated pregnancies were distributed according to the duration of pregnancy as follows: 84.9% under 12 weeks, 12.8% during 12–19 weeks and 2.3% during 20–23 weeks. In control women, the distribution was 89.8%, 8.7% and 1.5%, respectively (*p* = 0.03 for difference between women with and without type 1 diabetes).

### Indications for termination of pregnancy

**Fig. 2:**
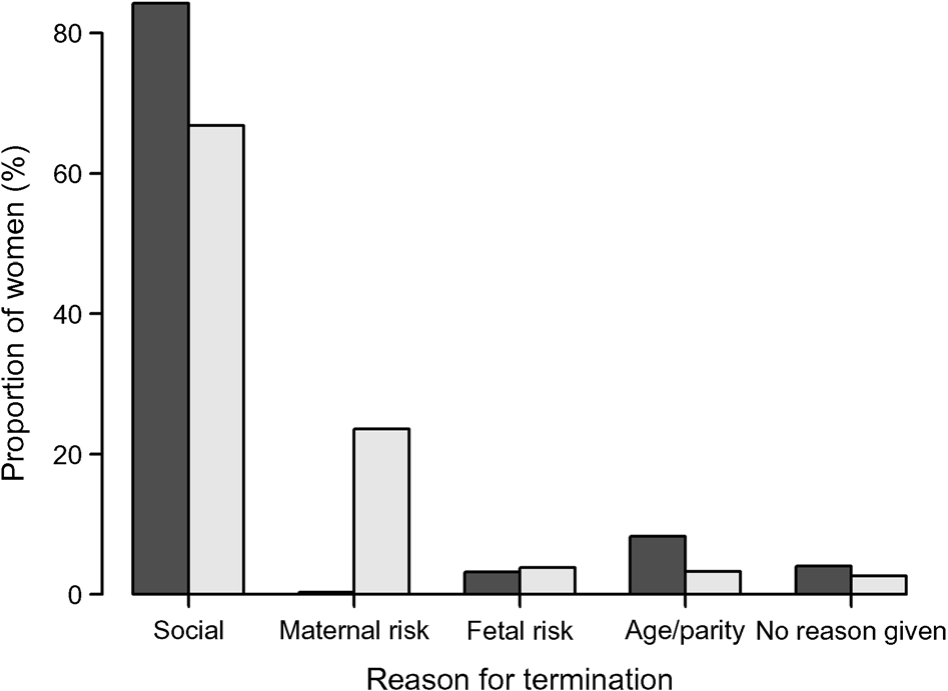
Numbers and indications of terminations of pregnancy in women with and without type 1 diabetes. Grey bars, with type 1 diabetes; black bars, without type 1 diabetes

We explored the indications for termination of pregnancy stated on the application forms: the frequencies of different indications differed between women with and without type 1 diabetes. There was a significant difference between the groups in distribution of termination indications (*p* = 1.12 × 10^−35^) (Fig. 2 **)**. The difference was pronounced for social indications (66.9% in the diabetes group, 84.2% in the control group), maternal medical indications (women with type 1 diabetes, 23.6%; control participants, 0.3%) and age- and parity-related indications (women with type 1 diabetes, 3.2%; control participants, 8.3%), whereas no significant difference was seen for suspected or diagnosed birth defects. In 38 application forms, data on indications were missing (9 women with diabetes and 29 control women).

### Repeated terminations of pregnancy

Of women with type 1 diabetes, 52 had more than one termination during the study period, with 116 terminations in this group in total; some women had more than two terminations. The distribution of pregnancy duration among these women was 93%, 6% and 1% for duration under 12 weeks’, 12–19 weeks’ and > 20 weeks’ gestation, respectively. There were relatively more social indications among the terminations performed in women with repeated procedures (83%) and the proportion of maternal medical indications was slightly lower (17%) than in the diabetes group as a whole. The proportions of fetal indications (suspected or diagnosed birth defects), age and parity indications were similar to the overall proportions in the diabetes group. In women without diabetes, there were 111 women with repeated terminations of pregnancy and they had 255 terminations. Here, the proportion of procedures performed before 12 weeks’ gestation was slightly higher (93%) and the proportion of social indications was larger (87%) than in the non-diabetes group as a whole.

### Sterilisation

**Fig. 3:**
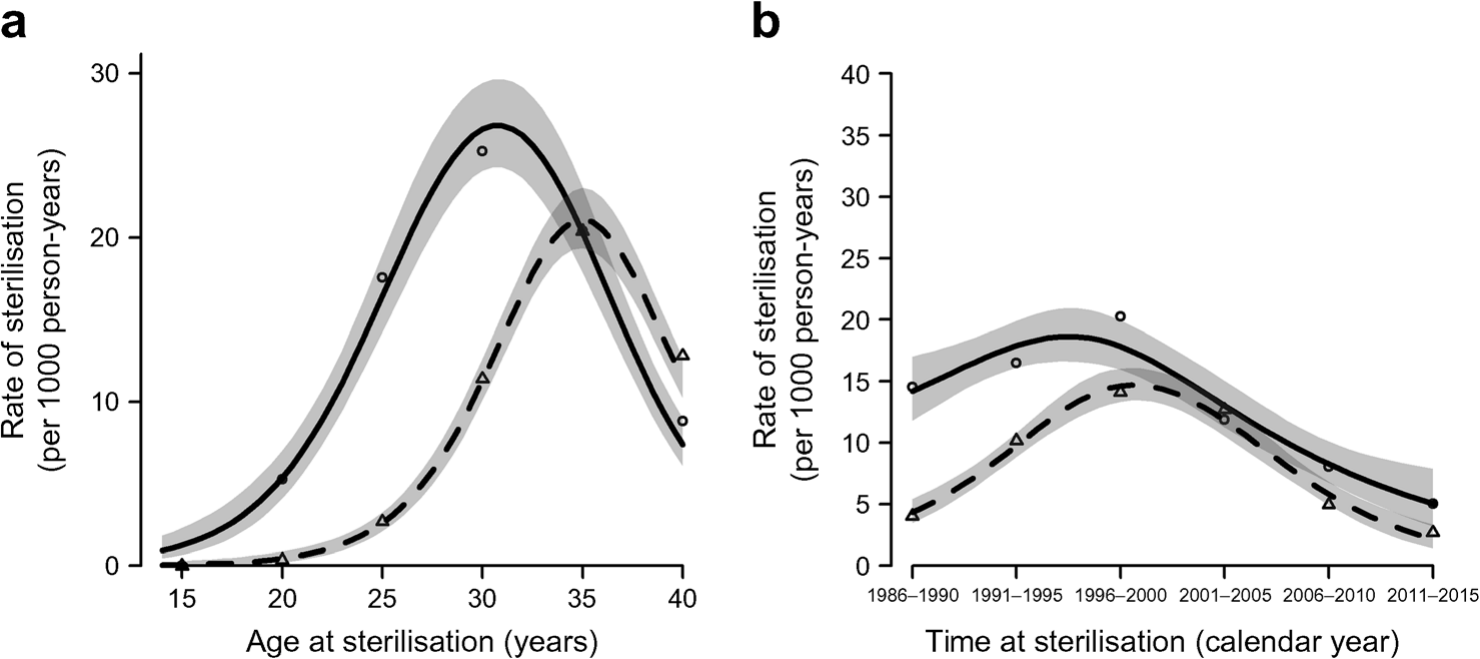
Sterilisation rates per 1000 person-years: (**a**) by age (*p* < 2.2 × 10^−16^); and (**b**) by calendar time (*p* = 1.544 × 10^−8^). The *p* values are for the difference between women with and without diabetes; 95% CIs are shown as shading. Solid line with circles, with type 1 diabetes; dashed line with triangles, without type 1 diabetes

Sterilisation was more common in women with type 1 diabetes (597 sterilisations in 2281 women [26.2%]) than in control women (873 sterilisations, 4509 women [19.4%]) (*p* = 1.50 × 10^−10^ for difference, *χ* ^2^ test); SIR 1.69 (95% CI 1.56, 1.83). The median age at the time of sterilisation in type 1 diabetes women was lower, at 31.1 years, than in control women, at 35.7 years (*p* = 2.20 × 10^−16^) (Fig. 3). The risk of sterilisation for women with type 1 diabetes was greater than for control women before the year 2000 (*p* = 1.48 × 10^−6^), but not later (Fig. 3).

Looking at sterilisations performed in women under 30 years of age, there were 141 operations in women with type 1 diabetes and 44 among women without type 1 diabetes. Of these, 27 were performed in women who had no children: 26 with type 1 diabetes and one in the control group.

In women with type 1 diabetes, 22.9% of all sterilisations were performed because pregnancy—or an additional pregnancy—was considered hazardous for the woman. Only 0.5% of control women underwent sterilisation with this indication (*p* = 2.20 × 10^−16^). Indications related to age and/or parity accounted for 99.3% of the sterilisations performed in control women, whereas in women with type 1 diabetes the proportion was significantly lower at 82.0% (*p* < 2.20 × 10^−16^).

## Discussion

In this population-based study, both termination of pregnancy and sterilisation were significantly more common in women with type 1 diabetes than in women without. The indications for both procedures also differed between these groups of women. Previously [20], we reported that people with childhood-onset type 1 diabetes had fewer live-born children than people without diabetes. The unique and high-quality registry data available in Finland enable this type of study to be carried out. The current findings add further information to the picture of reproductive health in women with type 1 diabetes.

The frequencies of different indications for termination of pregnancy differed between women with and without type 1 diabetes. Most importantly, in the type 1 diabetes group, 23.6% of all terminations were performed on the basis of maternal medical indications, whereas the corresponding frequency in the control group was only 0.3%. Additionally, 66.9% (women with type 1 diabetes) and 84.2% (control participants) of terminations were performed for social reasons. According to national statistics, terminations of pregnancy in Finnish women were performed for social reasons in 96.6%, for fetal indications in 2.2% and for maternal medical reasons in 0.6% of cases in 1987–2011 [21]. In our control group, the proportions of different termination indications were in line with the nationwide statistics.

There were more terminations of pregnancy in women with type 1 diabetes than in control women. In calculating the SIRs of terminations, we considered that women with type 1 diabetes would probably be less likely to get pregnant [20] and would therefore be considered to be at a lower risk of termination of pregnancy.

The women in our study cohort were born during 1947–1979 and our study covers registry data from 1987 to 2011. This means that many of the terminations of pregnancy and sterilisations performed in women in the cohort have not been included, as they would have been performed before the start of our study. The start date coincides with the establishment of the Finnish Birth Register; information from before 1987 is not as easily accessible. Because sterilisation is more often performed at a younger age in women with type 1 diabetes than in those without, this study probably underestimates the number of sterilisations in women with diabetes to a higher degree than in the women without diabetes. The number of terminations of pregnancy is also underestimated for many of the older women in the cohort, but probably more equally in the two groups of women.

The number of terminations of pregnancy in women with type 1 diabetes decreased before the year 2000 and then increased again, which cannot be explained by any changes in legislation or guidelines during this period. This probably mostly relates to the age distribution in the cohort; during the late 1980s and 1990s, a large proportion of women in the cohort were in their 30s and gave birth. In our study, the women with diabetes seldom had children after the age of 40, whereas the women without diabetes had their children more evenly throughout their fertile years. It could also be related to women with diabetes having acquired diabetic complications after a longer disease duration and thus not wanting to risk their health by having another pregnancy. There are relatively few women born in the 1970s in our cohort, which makes it difficult to estimate how the general progress in preventing diabetes complications has influenced pregnancy outcome and pregnancy planning in different birth cohorts.

In a Danish questionnaire study, 17.9% of reported pregnancies in women with type 1 diabetes ended with termination [13]. This study included women aged 20–65 years, which means that many of them were still of reproductive age. No information on termination indications was available. In a region in Denmark, data concerning all pregnancies in women with type 1 diabetes were recorded for a 15 year period, during which 12.5% of the pregnancies were terminated. Of the terminations performed in this study population, 24% were performed for maternal medical reasons [15]. In a more recent British study involving primary-care records, the frequency of termination of pregnancy was 9.6% in pregnancies with pre-pregnancy type 1 diabetes. Of these, 67% were performed for non-medical reasons [14]. The proportion of all pregnancies terminated in our study resembles more the Danish than the British results. This might partly be because the British study is more recent than both our study and the Danish study: better pre-pregnancy care might reduce the number of terminations of pregnancy. The proportions of maternal medical indications are quite similar in all three studies where they have been recorded.

Although birth defects in general are much more common in pregnancies of women with pre-pregnancy type 1 diabetes [10], in this study we detected no difference between women with and without type 1 diabetes in the proportion of terminations of pregnancy carried out for fetal indications. This might be a result of the low absolute number of suspected and detected birth defects in our study. In a British hospital-based cohort study conducted from 1997 with 462 recorded pregnancies in women with type 1 diabetes, there were 24 terminations of pregnancy, of which nine were performed because of congenital anomalies [12]. The Scottish Diabetes in Pregnancy Group recorded 273 pregnancies during a 1 year period; of these, 20 ended in termination. Of these 20, six were performed because of fetal indications [14].

For some reason, data on termination indication was missing in 38 forms, nine for women with diabetes and 29 without. These women represent 2.6% of the women with diabetes and 4% of the control women, proportions that can be considered small. In the clinical situation, the forms have most probably been completed by interviewing the women, but we did not have access to hospital records and could therefore not complete our data.

In our study, sterilisations were performed at a younger age in women with type 1 diabetes than in non-diabetic women, therefore they also happened earlier in the follow-up period. It is not possible to find out from our registry-based data whether these differences, which are inherently correlated, were primarily driven by age or calendar time,

In our study, 13 women (five with diabetes and eight without) underwent two sterilisation operations, of which the first one was obviously not successful as they all subsequently became pregnant and either gave birth or had a termination of pregnancy before the second one. Some women, however, might have undergone a sterilisation reversal operation after the first sterilisation, but we lack information on sterilisation reversal operations. None of the pregnancies following sterilisation procedures was a result of infertility treatment.

In the 2010s, sterilisation at young age is seldom needed in women with type 1 diabetes. Most contraceptive methods are nowadays considered suitable for women with diabetes and genuine family planning is very important to reduce the risk of birth defects. This includes providing information to all fertile-aged women with diabetes on folic acid supplementation before pregnancy, the importance of not smoking and achieving good metabolic control before conception, and factual research-based information on the risks that are still associated with pre-pregnancy diabetes. A further improvement in the clinical management of women with childhood-onset type 1 diabetes could also reduce the number of terminations of pregnancy that are performed for maternal medical indications.

During recent years, the frequencies of sterilisation in women with and without diabetes have been similar. The difference between the groups in our study vanished around the year 2000. Attitudes towards pregnancy in type 1 diabetes have changed during the long period covered by our study, and this might have affected the trends observed over time. Simultaneously, sterilisation as contraception has declined in popularity among women both with and without diabetes [22]. This change might be because of the increasing use of other efficient but reversible contraception methods, particularly hormonal intrauterine devices [21], which can be used in all women with or without diabetes.

In conclusion, the indications for termination of pregnancy and sterilisation are different in women with diabetes compared with other women. Pregnancies in women with type 1 diabetes are still terminated more often than in women without diabetes, but the difference in sterilisation rates has disappeared during recent years.

## Electronic supplementary material

ESM Methods (PDF 64.5 kb)

## Abbreviation

SIR: Standardised incidence ratio
