# Supplementary material for: Termination of pregnancy and sterilisation in women with childhood-onset type 1 diabetes
Source: Diabetologia. 2017 Sep 11;60(12):2377–83. doi: 10.1007/s00125-017-4428-7 (PMC6448903; doi:10.1007/s00125-017-4428-7)
Supplement: Supplementary file 1 — (PDF 64.5 kb) [file 125_2017_4428_MOESM1_ESM.pdf]

ESM Methods

Selection of the Finnish DERI cohort and the control persons

The patients in the DERI study were diagnosed with type 1 diabetes at 17 years of age or under during 1965–1979. They were placed on insulin at diagnosis and were residing in Finland. In the register, 5166 cases (2327 women) were identified with virtually complete case-ascertainment.

From the Central Population Register, two control persons without diabetes for each case were identified and matched for sex, year of birth and geographical birth region with the women with type 1 diabetes in the DERI cohort. Control women who had been discharged from hospital with a diagnosis of diabetes mellitus (according to the information from the HDR) were excluded. Our database was also used to investigate the incidence of diabetes among offspring, and thus the control women who had offspring with a diabetic spouse could also be excluded.
